# Comparison of efficacy of single-port versus conventional laparoscopic treatment for uterine leiomyoma: a latest meta-analysis

**DOI:** 10.3389/fonc.2023.1192582

**Published:** 2023-08-02

**Authors:** Yanhui Li, Rui Liu, Xue Li

**Affiliations:** ^1^ Department of Obstetrics and Gynaecology, Qilu Hospital of Shandong University, Jinan, Shandong, China; ^2^ Department of Obstetrics and Gynecology, Dezhou United Hospital, Dezhou, Shandong, China; ^3^ Department of Laboratory Medicine, People’s Hospital of Linyi County, Dezhou, Shandong, China

**Keywords:** single-hole laparoscopic, uterine leiomyoma, operation time, blood loss, cosmetic effect

## Abstract

**Objective:**

Single-port laparoscopy has been proposed as an ideal surgical method for the treatment of uterine leiomyoma. It can effectively remove the lesion, reduce the loss of hemoglobin, and has superior cosmetic effects. Therefore, we searched relevant studies and conducted a meta-analysis to evaluate the effect of single-port laparoscopy on myoma resection, hemoglobin loss, and scar beauty compared to conventional laparoscopy.

**Methods:**

We systematically searched PubMed, EMBASE, scope, Cochrane, CNKI, and other databases to find randomized controlled studies on the efficacy of single-port laparoscopy and traditional laparoscopy for meta-analysis. The main outcomes of our study were the duration of surgery, the reduction of hemoglobin, and the cosmetic effect of the postoperative scar. The effect model was selected according to heterogeneity (random effect model or fixed effect model), and the relevant sensitivity analysis and publication bias test were performed.

**Results:**

We searched a total of 501 related literature articles and finally included 19 studies involving 21 researchers. Comparison of single-port laparoscopic myomectomy with traditional surgery: Operation time had no significant difference (Standardized Mean Difference [SMD]: 0.13, 95% Confidence interval (CI), -0.04 to 0.30; I²=74%; P = 0.14); The reduction of hemoglobin is lower ([SMD]: -0.04; 95% CI, -0.23 to 0.14; I²=71%; P = 0.65), and the cosmetic effect of postoperative scar is more satisfactory ([SMD]: 0.42, 95% CI: 0.02 to 0.83; I²=72%, P= 0.04). There was no significant difference in conversion rate, postoperative pain, blood loss, postoperative gastrointestinal recovery time, or length of hospital stay.

**Conclusion:**

Compared with traditional laparoscopy, the operation time of the treatment of uterine leiomyoma by single-port laparoscopy is not extended, the reduction of hemoglobin is less, and the cosmetic effect of the scar is better. Therefore, single-port laparoscopy is superior to traditional surgery in the treatment of uterine leiomyoma.

**Systematic review registration:**

https://inplasy.com/inplasy-2023-3-0071/, identifier INPLASY202330071.

## 1 Introduction

Uterine leiomyoma is the most common benign disease of the female reproductive tract, which is associated with abnormal uterine bleeding (increased menstruation, secondary anemia), pelvic compression symptoms (abnormal urination, constipation, and diarrhea), recurrent miscarriage, and low fertility (1, 2). Surgery is still the main strategy for uterine leiomyoma (3, 4). With the development of medical technology and the spread of minimally invasive ideas, single-port laparoscopy is increasingly applied in gynecological surgery. Compared with traditional multiport laparoscopy, it has the advantages of satisfactory operation effect, less intraoperative hemoglobin loss, excellent cosmetic effect, and postoperative complications (5). However, the limited space for the instruments to move and the interference between each other increase the difficulty of operation, so the single port laparoscopic is not widely used (6, 7). In the process of hysteromyoma resection and uterine wall defect sutures, multiple sutures need to be bound by single-port laparoscopy. Especially in the process of suture and knot, sutures with sufficient tension can avoid the extension of operating time and the increase of intraoperative blood loss (8, 9). These surgical procedures increase the difficulty of single-port laparoscopic myomectomy and relatively limit its widespread implementation. Nevertheless, the advantages of robotic single-port laparoscopic myomectomy include stable three-dimensional vision, wrist instruments, tremor elimination software, precise anatomy, and easier knotting and suturing (10–13). In recent years, robotic single-port laparoscopic myomectomy has been performed. Although, it also has a long learning curve and the engineering burden of relatively unskilled coordination of the arm with the accessory ports of the assistant (14). However, clinical studies suggest that it has the advantages of satisfying surgical and cosmetic effects, less intraoperative blood loss and postoperative complications, shorter hospital stay, and lower pain score. Given the limited level of evidence provided by a single clinical study, we conducted this meta-analysis to compare the efficacy of single-port laparoscopic myomectomy with conventional laparoscopic surgery.

## 2 Materials and methods

This meta-analysis strictly complies with the Preferred Reporting Project (PRISMA) statement and the MOOSE (Meta-analysis of Observational Studies in Epidemiology) statement and is registered in the International Platform of Registered Systematic Review and Meta-analysis Protocols (INPLASY202330071).

### 2.1 Search strategy

We searched EMBASE, PubMed, Cochrane, Web of Knowledge, Cochrane, China National Knowledge Infrastructure, and other databases (published until December 2022) to compare the effectiveness and safety of single-port laparoscopic hysterectomy with that of multiport laparoscopy hysterectomy. The literature we searched was not restricted by language. We used the following combination of text and MeSH terms: (“single port” and “uterine leiomyoma”). The complete retrieval used for PubMed is “single port” [MeSH term] or “single incision” [text word] or “unit point” [text word] and “uterine leiomyoma”[text word] or “uterine fibroids” [text word] or “robot” [text word]. We considered all potentially eligible studies for the review, regardless of the primary outcome. We also performed a manual search with a reference list of key articles published in English.

### 2.2 Study selection

The inclusion criteria were: (1) Controlled clinical study; (2) Patients with uterine leiomyoma receiving single-hole laparoscopic laparoscopy or conventional laparoscopy; (3) Types of intervention, the study should compare the treatment of uterine leiomyoma by single-port laparoscopy with traditional laparoscopy. If single-port laparoscopy was not the primary intervention but controlled conventional laparoscopy therapy with the addition of auxiliary ports, this study would have been excluded; (4) Outcomes: Studies should measure at least one of the outcomes.

Exclusion criteria: (1) Serial case reports, editorials, letters to editors, review articles, case reports, or animal experimental studies; (2) Two or more studies reported by the same author or center; (3) Data from single multicenter studies involving other studies; (4) No outcome data to study from reported results; (5) If single-hole laparoscopy control laparoscopy does not involve the outcome of uterine leiomyoma.

### 2.3 Data extraction

We compared the treatment of uterine fibroids with single-port laparoscopy versus traditional laparoscopy: Primary outcomes were operative time (total operative time from the first skin incision to skin closure at the last port site), amount of hemoglobin change (postoperative decrease in hemoglobin level was defined as the difference between the hemoglobin level on the morning of surgery and the hemoglobin level on the day one after surgery), estimated blood loss, pain scores [measurement of postoperative pain by visual analog scale (VAS) and analgesic need]and aesthetic satisfaction (measurement of aesthetic satisfaction based on scales given to patients 30 days after surgery). Secondary outcomes were the length of hospital stay, time to first gastrointestinal activity (from the end of anesthesia to the first occurrence of intestinal gas passages), conversion rate (addition of the puncture device, or conversion to laparotomy), blood transfusion, fever (temperature ≥38°C, two consecutive times at least six hours apart, except the previous 24 hours), and time to first postoperative walk.

### 2.4 Quality assessment of the included studies

Eligible study titles and abstracts were examined by two independent investigators (RL and XL) who read the full text for evaluation. They extracted the data and conducted a detailed analysis, and if controversial in the process, sought a third researcher (YL) to resolve the problem. We extracted the following data from the included studies: authors, year of publication, total patients, age, type, study, controls, body mass index, surgery, time, conversion, blood loss, hemoglobin, postoperative pain, hospitalization, bowel recovery, first postoperative walk and complications (postoperative anemia, blood transfusion, fever, and partial port infection and intestinal obstruction). Two independent evaluators (RZ and YL), assessed the risk of bias following the PRISMA recommendations.

### 2.5 Data synthesis and analysis

We evaluated the impact of single-port laparoscopy versus conventional laparoscopy myomectomy outcomes. All meta-analysis data were performed by review manager 5.4 (Cochrane Collaboration Network, Oxford, UK). SMD (standardized mean difference) and odds ratio (OR) are employed for continuous and binary variables, respectively. All results were recorded with 95% confidence intervals (CI). Heterogeneity between studies was assessed with I², if I²≤50%, a fixed-effect model was used; I² > 50%, which is considered to have great heterogeneity, the random effects model is applied. Sensitivity analysis estimates the effect of high-bias risk studies on the overall effect. Begg’s and Egger’s test evaluated whether there was potential publication bias.

## 3 Results

### 3.1 Characteristics of included studies

**Figure 1:**
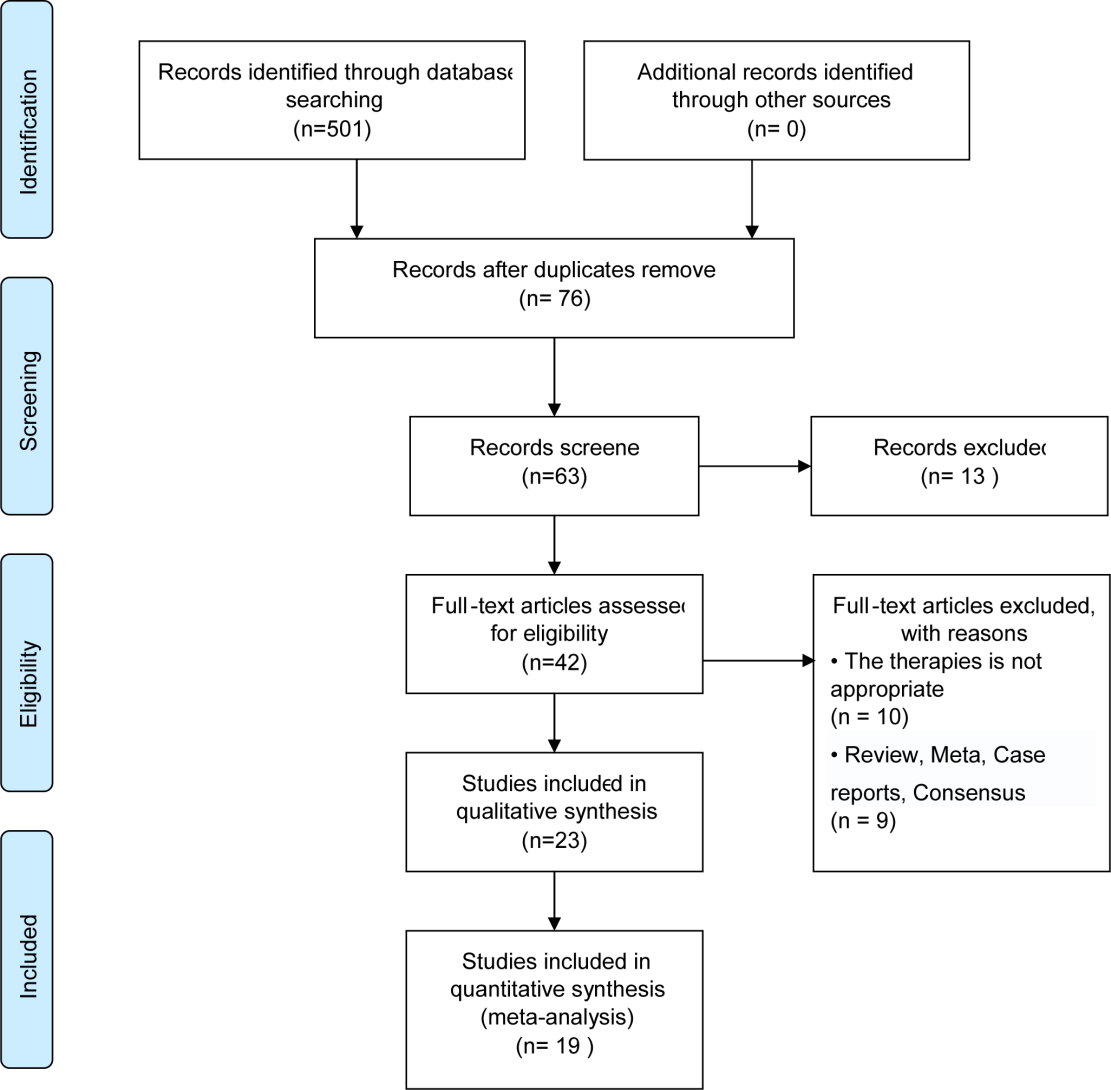
Flow diagram of literature search and data extraction.

A total of 501 studies were included, including 19 studies (with data from 3352 patients) (**Figure 1**) (15–33). They were published between 2013 and 2022 (**Table 1**, **Supplement Table 1**). Five studies were from China, one from the United States, and 13 from South Korea. Two of the studies involved multiple centers. Five studies compared operative time, the reduction in hemoglobin, blood loss, pain score, and cosmetic outcomes of robotic and conventional laparoscopic single-port surgery. All of the included studies claimed in the disclosure of interest that all authors had nothing to disclose and did not report any potential conflicts of interest.

**Table 1:** Table of baseline characteristics included in the study.

Author	Year	Country	Study type	Experimental groups	Control groups	No. patients		Age (years)		BMI	
				LESS	MPL	LESS	MPL	LESS	MPL	LESS	MPL
Ji Hye Kim (20)	2022	Korea	retrospective case-control	single-site robotic myomectomy (SSRM)	laparoscopic myomectomy (LM)	46	46	39.37±6.71	39.5±7.43	22.5(20-26.25)	23(21-25.25)
SuHyeon Choi (16)	2019	Korea	retrospective case-control	robotic single-site myomectomy (RSSM)	robotic multi-site myomectomy (RMSM)	105	105	36.7±6.3	37.2±5.6	21.6±3.2	22.5±3.2
SoHyun Ahn (15)	2021	Korea	retrospective case-control	robotic single-site (RSS)	conventional robotic multi-port	90	90	36.1±6.5	37±5.8		
Taejong Song (29)	2015	Korea	Prospective randomized controlled trial	laparoendoscopic single-site myomectomy	conventional laparoscopic myomectomy	50	50	39.1±5.9	39.4±5.5	22.7±4.1	22.3±2.5
Tae-JoongKim (24)	2015	Korea	Prospective randomized controlled trial	laparoendoscopic single-site surgery	laparoendoscopic multiport surgery	125	126	47 (28-60)	47 (35-54)	23.4 (17.1-35.8)	23.4 (18.0-33.3)
Chien-Min Han (18)	2013	China	retrospective case-control	single-port laparoscopic myomectomy (SPLM)	traditionallaparoscopic myomectomy (LM)	10	10	45.5 (35.5-50.3)	41 (32.5-49.3)	24.7 (21.2-27.7)	23.3 (20.8-28.3)
Gaby N.Moawad (28)	2019	USA	multileft retrospective analysis	robotic single-site myomectomy (RSSM)	robotic multiport myomectomy (RMM)	80	95	39.1 (6.05)	36.1 (5.7)		
Seul KiKim (22)	2014	Korea	retrospective case-control	laparoendoscopic single-site myomectomy (LESS-M)	conventional laparoscopic myomectomy (CLM)	59	59	41.5±5.5 (30–52)	41.2±5.3 (25–53)	22.8±2.6(17.8-27.7)	22.4 ± 3.(17.9-30.6)
Ji YeKim (21)	2014	Korea	Prospective matched case-control	laparoendoscopic single-site myomectomy (LESS-M)	conventional laparoscopic myomectomy (LM)	45	90				
DayongLee (25)	2018	Korea	Prospective randomized controlled trial	single-port laparoscopic myomectomy (SP-LM)	Conventional Laparoscopic Myomectomy	30	29	41.1±6.5 (27–56)	41.4±5.6 (30–51)	23.4±3.6 (18.2-32.8)	23.3±3.4(17.9-30.1)
SuMi Kim (23)	2015	Korea	retrospective case-control	Single port Laparoscopic Myomectomy	TwoPort Laparoscopic Myomectomy	61	37	39.6±7.1	38.5±7.7	24.1±4.2	23.0±3.6
JongMinBaek (17)	2015	Korea	retrospective case-control	Single port Laparoscopic Myomectomy	Three-Port Laparoscopic Myomectomy	61	93	39.6±7.1	39.6±7.7	24.1±4.2	23.5±3.7
Shi-Fang Zhou (32)	2021	China	retrospective case-control	single-site myomectomy (LESS-M)	conventional laparoscopic myomectomy	233	233	39.8±6.2	40.0±6.6	22.1±1.9	22.0±3.0
Sa-Ra Lee (26)	2021	Korea	retrospective case-control	single-incision robotic myomectomy	multiport robotic myomectomy	58	148	35.91±5.74	35.14±6.28	21.5±3.30	21.64±2.98
Ju-Hee Kim (26)	2021	Korea	retrospective case-control	single-incision robotic myomectomy	multiport robotic myomectomy	59	403	34.97 ±6.37	37.99±5.77	21.58±3.28	22.59±3.67
Lili Jiang (19)	2021	China	retrospective case-control	single-port laparoscopy	traditional three-port laparoscopy	60	60	38.10 ± 6.47	37.17± 6.54		
Wei Zhu (33)	2022	China	retrospective case-control	laparoendoscopic single-site myomectomy (LESS-M)	conventional multiport laparoscopic myomectomy (MLS-M)	35	30	29.26 ±5.18	28.6±4.60	23.1±1.64	22.9± 1.45
Ying Zhang (31)	2021	China	Prospective randomized controlled trial	laparoscopic single-site surgery (LESS)	conventional laparoscopy (CL)	5	6	34.5±5.5	35.5±5.2	23.6 ±4.5	22.6 ±4.8
Jeong MinEom (17)	2013	Korea	Prospective case-control	laparoendoscopic single-site myomectomy (LESS-M)	conventional laparoscopic myomectomy (LM)	65	65	43.98 ± 10.70	46.18 ± 9.72	23.67±3.06	23.7± 3.86
Suk Woo Lee (27)	2017	Korea	Prospective randomized controlled trial	single-port (SP) laparoscopic surgery	multiport laparoscopic myomectomy (MP-LM)	100	69	43.1±6.2 (24-56)	41.6±6.0 (27-52)	23.2±2.8 (18.4-30.1)	22.6±2.3 (18.7-28.6)
Jin-SungYuk (30)	2015	Korea	Prospective randomized controlled trial	single port laparoscopic myomectomy	Single-port laparoscopically assisted-transumbilical ultraminilaparotomic myomectomy (SPLA-TUM)	46	46	36.9±6.8	37.3±6.3	22.9±3.3	21.7±2.8

No. patients, Number of patients; LESS, laparo-endoscopic single site surgery; MPL, multiple-port laparoscopy.

### 3.2 Methodological quality

We used Cochrane’s grade risk assessment system for bias risk analysis (**Table 2**). There was no high risk of bias in our inclusion. Six studies reported sufficient randomization because single-port laparoscopy and conventional laparoscopy failed to perform blinding and included studies did not perform adequate assignment concealment.

**Table 2:** GRADE assessment of risk bias in included studies.

Interventions for [Condition] in [Population]							
Outcomes	Intervention and Comparison intervention	Illustrative comparative risks* (95% CI)		Relative effect (95% CI)	No of Participants (studies)	Quality of the evidence (GRADE)	Comments
		Assumed risk	Corresponding risk				
		With comparator	With intervention				
Single port laparoscopic treatment of uterine fibroids							
	Time of operation/[Uterine fibroids]		The mean single port laparoscopic treatment of uterine fibroids in the intervention groups was **0.13 standard deviations higher** (0.04 lower to 0.3 higher)		2753 (17 studies)	⊕⊕⊝⊝ **low**	SMD 0.13 (-0.04 to 0.3)
Blood loss							
	Blood loss/[Uterine fibroids]		The mean blood loss in the intervention groups was **0.25 standard deviations lower** (0.73 lower to 0.22 higher)		2492 (15 studies)	⊕⊝⊝⊝ **very low**	SMD -0.25 (-0.73 to 0.22)
Hemoglobin							
	Hemoglobin/[Uterine fibroids]		The mean hemoglobin in the intervention groups was **0.04 standard deviations lower** (0.23 lower to 0.14 higher)		2158 (11 studies)	⊕⊕⊝⊝ **low**	SMD -0.04 (-0.23 to 0.14)
Pain score							
	6h Pain score/[Uterine fibroids]		The mean pain score in the intervention groups was **0.01 standard deviations higher** (0.23 lower to 0.25 higher)		271 (3 studies)	⊕⊕⊕⊝ **moderate**	SMD 0.01 (-0.23 to 0.25)
24h Pain score							
	24h Pain score/[Uterine fibroids]		The mean 24h pain score in the intervention groups was **0.01 standard deviations lower** (0.19 lower to 0.17 higher)		1186 (7 studies)	⊕⊕⊝⊝ **low**	SMD -0.01 (-0.19 to 0.17)
48h Pain score							
	48h Pain score/[Uterine fibroids]		The mean 48h pain score in the intervention groups was **0.13 standard deviations lower** (0.3 lower to 0.04 higher)		541 (4 studies)	⊕⊕⊕⊕ **high**	SMD -0.13 (-0.3 to 0.04)
Hospital stays							
	Hospital stays/[Uterine fibroids]		The mean hospital stays in the intervention groups was **0.32 standard deviations lower** (0.63 to 0.01 lower)		1280 (9 studies)	⊕⊕⊝⊝ **low**	SMD -0.32 (-0.63 to -0.01)
Recovery of intestinal tract							
	Recovery of intestinal tract/[Uterine fibroids]		The mean recovery of intestinal tract in the intervention groups was **2 standard deviations lower** (4.68 lower to 0.69 higher)		754 (5 studies)	⊕⊝⊝⊝ **very low**	
VAS score							
	VAS score/[Uterine fibroids]		The mean vas score in the intervention groups was **1.03 higher** (0.53 to 1.53 higher)		271 (3 studies)	⊕⊕⊝⊝ **low**	
Scar VAS score							
	Scar VAS score/[Uterine fibroids]		The mean scar vas score in the intervention groups was **0.42 standard deviations higher** (0.02 to 0.83 higher)		352 (3 studies)	⊕⊕⊝⊝ **low**	SMD 0.42 (0.02 to 0.83)
Blood transfusion							
	Blood transfusion/[Uterine fibroids]	**Study population**		**OR 0.78** (0.45 to 1.36)	692 (4 studies)	⊕⊕⊕⊕ **high**	
		**94 per 1000**	**75 per 1000** (44 to 123)				
		**Moderate**					
		**72 per 1000**	**57 per 1000** (34 to 95)				
Fever							
	Fever/[Uterine fibroids]	**Study population**		**OR 0.95** (0.58 to 1.57)	1366 (5 studies)	⊕⊕⊝⊝ **low**	
		**114 per 1000**	**109 per 1000** (69 to 168)				
		**Moderate**					
		**22 per 1000**	**21 per 1000** (13 to 34)				
First walk after surgery							
	First walk after surgery/[Uterine fibroids]		The mean first walk after surgery in the intervention groups was **0.6 standard deviations lower** (0.9 to 0.31 lower)		185 (2 studies)	⊕⊝⊝⊝ **very low**	SMD -0.6 (-0.9 to -0.31)
Transition							
	Transition/[Uterine fibroids]	**Study population**		**OR 3.8** (1.25 to 11.6)	1010 (7 studies)	⊕⊕⊝⊝ **low**	
		**6 per 1000**	**21 per 1000** (7 to 62)				
		**Moderate**					

### 3.3 Primary outcome

**Figure 2:**
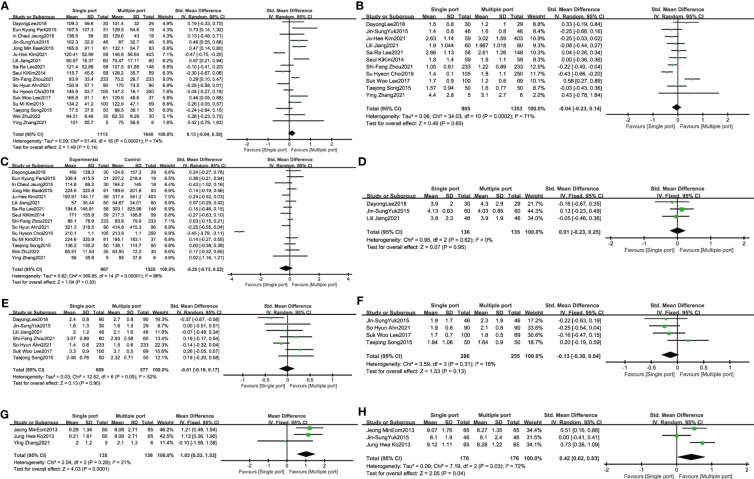
The primary outcome of single port laparoscopic myomectomy compared with traditional laparoscopic surgery **(A)**. Operative time **(B)**. Hemoglobin change **(C)**. Blood loss **(D)**. 6-hour pain scores **(E)**. 24-hour pain scores **(F)**. 48-hour pain scores **(G)**. Patients’ overall satisfaction with surgery **(G)**. Aesthetic satisfaction.

Data from 17 studies evaluating the comparison of single-port laparoscopic (N=1113) versus conventional laparoscopy (N=1640) showed the operation time did not extend (SMD: 0.13, 95% CI: -0.04 to 0.30; I²=74%, P= 0.14) (**Figure 2**). The quantity has not changed in postoperative hemoglobin measurements between single-port laparoscopic and conventional laparoscopic uterine leiomyomectomy (2158 patients in 11 studies, SMD: -0.04, 95% CI: -0.23 to 0.14; I²=71%, P= 0.65). There was no increase in blood loss in the single-port laparoscopic group compared with conventional surgery (15 studies, 2492 patients, SMD: -0.25, 95% CI: -0.73 to 0.22; I²=96%, P= 0.30). No significant difference was observed in pain assessment. (six hour pain assessment: N= 271, SMD: 0.01, 95% CI: -0.23 to 0.25; I²=0%, P= 0.95; 24-hour pain assessment: N= 1186, SMD: -0.01, 95% CI: -0.19 to 0.17; I²=52%, P= 0.90; 48-hour pain assessment: N= 541, SMD: -0.13, 95% CI: -0.30 to 0.04; I²=16%, P= 0.13). Evaluation of the cosmetic effect of postoperative scar recovery showed that the cosmetic effect of myomectomy under single-port laparoscopy was significantly better than that under traditional laparoscopy (N= 352, SMD: 0.42, 95% CI: 0.02 to 0.83; I²=72%, P= 0.04) (**Figure 2**).

### 3.4 Secondary outcomes

**Figure 3:**
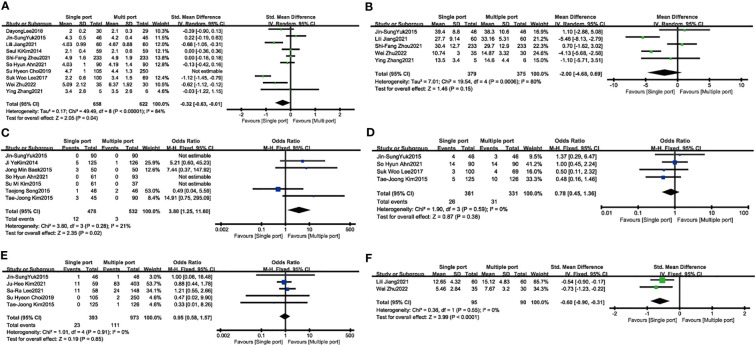
The secondary outcome of single port laparoscopic myomectomy compared with traditional laparoscopic surgery **(A)**. The length of hospital stay **(B)**. Time to first gastrointestinal activity **(C)**. Conversion rate **(D)**. Blood transfusion **(E)**. Fever **(F)**. Time to first postoperative walk.

Fourteen studies reported length of hospital stay and four were excluded because the data were expressed as median (interquartile distance). Data from 10 studies involving 1635 patients showed no significant difference in length of hospital stay between single-port and conventional laparoscopy (SMD: -0.25; 95%CI, -0.54 to 0.04; P = 0.09) (**Figure 3**). In gastrointestinal recovery time, five studies reported included 754 patients with approximately similar time between the two groups (SMD: -0.41; 95%CI, -0.94 to 0.12; P= 0.13) (**Figure 3**). Single-port laparoscopic myomectomy with additional puncture devices or conversion to laparotomy was significantly less than in the traditional groups (N= 1010, SMD: 3.80, 95% CI: 1.25 to11.60; I²=21%, P =0.02) (**Figure 3**). Four studies, including 692 patients, showed that transfusions of blood products were comparable between the two groups (N= 352, OR: 0.78, 95% CI: 0.45 to 1.36; I²=0%, P= 0.38) (**Figure 3**). Five studies suggested that postoperative fever was almost the same in the two groups. (N= 1366, OR: 0.95, 95%CI: 0.58 to 1.57; I²=0%, P= 0.85) (**Figure 3**). Two studies compared the first postoperative walk time in the single-hole surgery group with that in the conventional laparoscopic group, suggesting earlier movement out of bed after single-hole surgery (N= 185, SMD: -0.60, 95% CI: -0.90 to -0.31; I²=0%, P < 0.001) (**Figure 3**).

### 3.5 Sensitivity analysis

**Figure 4:**
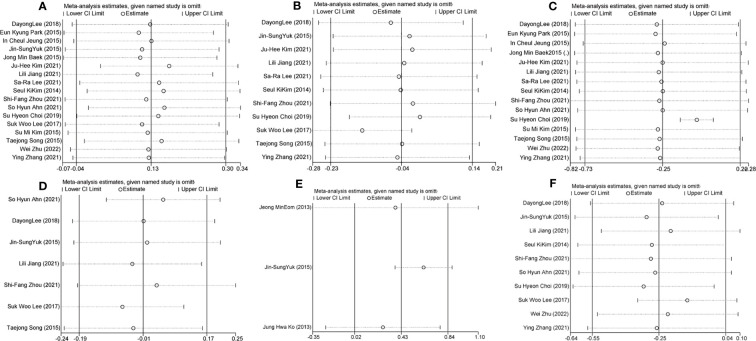
Sensitivity analysis of the comparative study of efficacy between single-port laparoscopy and conventional laparoscopy with the leave-one-out method **(A)**. Hemoglobin reduction **(B)**. Blood loss **(C)**. 24-hour pain score **(D)**. Scar beauty satisfaction assessment **(E)**. Hospital stay.

We compared the efficacy of single-port laparoscopy with that of conventional laparoscopy and analyzed the sensitivity of studies with the leave-one-out method. The overall heterogeneity, effect size, and 95% CI of operation time did not change significantly after the leave-one-out method (**Figure 4**). In terms of hemoglobin reduction, heterogeneity, overall effect size, and 95% CI (SMD: -0.15, 95% CI: -0.28 to-0.02; I²=34%, P=0.03) were changed by eliminating one study [Suk Woo Lee, 2017 (27)]. When one study [Su Hyeon Choi, 2019 (16)] was removed successively in blood loss, the heterogeneity of the studies decreased significantly (I²=96% to 10%), but the overall effect size and 95% CI did not change extremely, indicating that the heterogeneity of the results of this study may come from this study (**Figure 4**). The results of the 24-hour pain scores did not change remarkably in terms of heterogeneity, overall effect size, and 95% CI after the removal of one study. The results of the scar beauty satisfaction assessment, excluding this study in the sensitivity analysis [Jin-Sung Yuk, 2015 (30)], showed a significant decrease in heterogeneity (SMD: 0.62, 95% CI: 0.37 to 0.87; I²=0%, P < 0.001), suggesting that this study may be the source of heterogeneity (**Figure 4**). Comparing the length of hospital stay for single-port and multi-port laparoscopy, the sensitivity analysis excluding one study [Su Hyeon Choi, 2019 (16)] suggested that the length of hospital stay for single-port laparoscopy was shorter than that of traditional laparoscopy (SMD: -0.32, 95% CI: -0.63 to -0.01; I²=34%, P=0.04) (**Figure 4**).

### 3.6 Publication bias analysis

**Figure 5:**
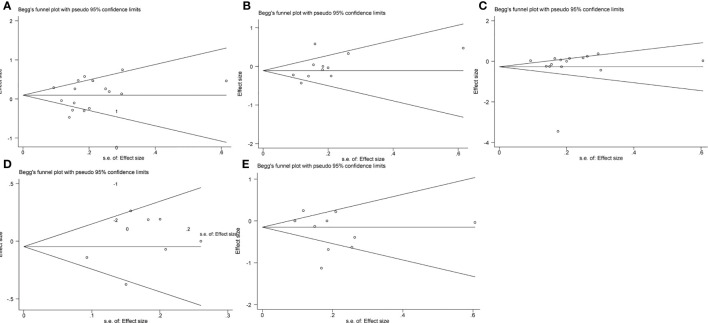
Publication bias analysis: Begg’s test **(A)**. The operation time **(B)**. Hemoglobin reduction **(C)**. Blood loss **(D)**. 24-hour pain scores **(E)**. Hospital stay.

**Figure 6:**
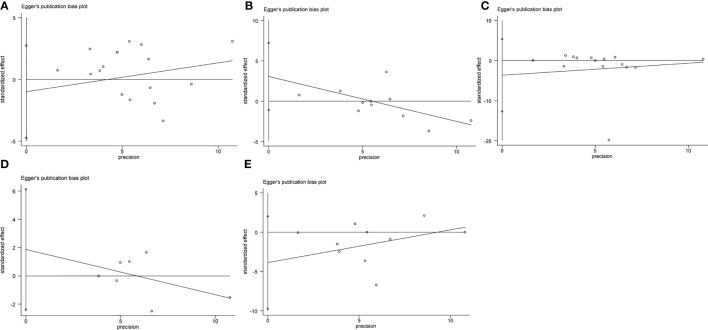
Publication bias analysis: Egger’s test **(A)**. The operation time **(B)**. Hemoglobin reduction **(C)**. Blood loss **(D)**. 24-hour pain scores **(E)**. Hospital stay.

We conducted the Begg’s and Egger’s test to evaluate the publication bias of the included single-port laparoscopy versus conventional laparoscopy myomectomy studies. Their analysis results are: the operation time (Begg’s test p=0.39; Egger’s test p=0.59), hemoglobin loss (Begg’s test p=0.12; Egger’s test p=0.13), blood loss (Begg’s test p=1.00; Egger’s test p=0.40), 24-hour pain scores (Begg’s test p=0.11; Egger’s test p=0.17) and hospital stay (Begg’s test p=1.00; Egger’s test p=0.30). These results indicate that they may be free of publication bias (**Figures 5**, **6**).

## 4 Discussion

### 4.1 Implications

Our results suggest that compared with traditional laparoscopy, single-port laparoscopy myomectomy has advantages such as no increased operation time, reduction in hemoglobin, blood loss, better cosmetic effects after scar recovery, shorter hospital stay, lower conversion rate, and earlier first postoperative activity. Meanwhile, there was no significant difference in pain score, gastrointestinal recovery, blood transfusion, and postoperative fever. Therefore, single-port laparoscopic myomectomy is a relatively safe, efficient, and ideal surgical method with satisfactory cosmetic effects. Laparoscopy surgery in obstetrics and gynecology arises with the characteristics of small trauma, less blood loss and complications, and fast recovery, which can promote the early recovery of patients (5, 34). The initial stage is mainly for the traditional three-hole and four-hole laparoscopic application in the removal of uterine leiomyoma. Now, with the deepening of the concept of minimally invasive surgery, single-port laparoscopy, and robotic single-port laparoscopy have been considered as a new clinical surgical strategy, with less invasive, faster postoperative recovery, and better aesthetic effects of safe and feasible hot surgical methods (9, 35, 36). At the same time, vNOTES combines traditional vaginal surgery with single-port laparoscopic surgery and has been shown to be a viable and safe approach. It has the advantages of less complications, less postoperative pain, quick recovery and short hospital stay (37). Unfortunately, at present, there are few comparative studies on vNOTES and transabdominal single-pore and single-pore laparoscopic myomectomy, and it is not possible to conduct an effective meta-analysis. In terms of safety, length of operation, length of hospital stay, blood loss, blood transfusions, and fertility outcomes, single-port laparoscopic myomectomy has been reported to produce favorable outcomes compared to laparotomy or laparoscopic hysterectomy. There is no denying that single-incision laparoscopic myomectomy is not ergonomic and requires the surgeon to be highly skilled in overcoming instrument congestion and limited depth perception. The type, size, and location of the fibroids can also be a challenge in performing single-hole laparoscopic myomectomy. For experienced gynecological surgeons, ergonomics has overlapping features in both traditional robotic surgery and laparoscopic surgery, requiring less time to adapt. The robotic surgical system has stable 3D vision, wrist function, elimination of tremors, higher precision anatomy for easier knotting and binding, and good ergonomics. In addition, single-port laparoscopic myomectomy avoids the risk of tumor spread caused by traditional laparoscopic dynamic uterine fragmentation to a certain extent. In single-port laparoscopic myomectomy, the extracted fibroids are placed in a sample bag, removed to the umbilical cord incision and removed with a cold knife to reduce the risk of fibroid spread. Although, the ports used in single-port laparoscopic surgery are expensive, increasing the cost of surgery. However, disposable, expensive instruments can be replaced with surgical gloves to mitigate collisions without the additional cost of laparoscopic single-site surgery (LESS). Homemade multichannel laparoscopic surgical gloves have the distinct advantages of being less costly and more readily available. Optimize the standardization of surgical methods, which will allow for meta-analysis and the ability to objectively compare surgical outcomes (38).

### 4.2 Strengths and limitations

In this study, we found that the reduction in the number of laparoscopy ports was no worse than conventional laparoscopy myomectomy in terms of surgical time and measured interpretative blood loss, despite difficulties such as interference from surgical instruments, poor vision, and a long learning curve (39). At the same time, sensitivity analysis excluded a study with large heterogeneous sources [Suk Woo Lee, 2017 (27)], and we found that the reduction of hemoglobin in myomectomy under single-port laparoscopy was less than that under conventional laparoscopy. Although this meta-analysis of pain scores comparing single-port versus conventional laparoscopy myomectomy at 6-hour, 24-hour, and 48 -hour did not show increased or less severe pain. This may be because visceral pain is similar to and stronger than skin incision pain (40). In addition, despite the reduced number of ports, stretching of the umbilical fascia and subsequent extension of the length of the skin incision appears to be inevitable due to the crowding and passage of laparoscopy instruments (35, 41). Moreover, in our meta-analysis, results were included in the vancouver scar scale (VSS), the observer scar assessment scale (OSAS), the patient and observer scar assessment scale (POSAS), and the visual analog scale (VAS)]. Compared with traditional laparoscopy uterine leiomyosarcoma, single-mouth laparoscopy surgery is characterized by “no scar”, more beautiful incision, improved patient satisfaction, faster postoperative recovery, and so on, because it uses the skin folds formed naturally in the navel as the channel to hide the surgical incision (42). In terms of length of hospital stay, although public health policies and customs of different countries may have a greater impact on the length of hospital stay, sensitivity analysis conducted by successively removing one study suggests that after removing the heterogeneous studies [Su Hyeon Choi, 2019 (16)], the length of hospital stay in the single-port laparoscopy myomectomy group is shorter than that in the traditional laparoscopy groups. The results were statistically obvious. In the transformation, there was a significant decrease in myomectomy under single-port laparoscopy compared with that under traditional laparoscopy, indicating that although single-port laparoscopy has narrow operating space, difficult positioning in space and distance, and high surgical difficulty, it does not increase puncture holes and conversion to laparotomy due to the difficult exposure of anatomical levels and the risk of excessive interpretative bleeding (43, 44). Finally, many studies have demonstrated that compared with conventional laparoscopic hysterectomy, single-port laparoscopic hysterectomy has fewer postoperative complications (blood transfusion, fever, first postoperative walking time, and postoperative gastrointestinal recovery time). However, the results of our meta-analysis may not show significant differences owing to the small number of studies that included relevant outcomes (45). Admittedly, there are limitations to our study: 1. First, the six studies included in this analysis were generated according to a random sequence, but did not adequately assign concealment and strictly implement subject and investigator blindness; 2. Secondly, there were only two multi-center cooperative studies in our retrieval, but the sample size of most studies was not substantial enough. Such insufficient data may result in a lack of statistical significance and heterogeneity; 3. It is not clear which number and type of fibroids and where the fibroids are located are more likely to result in better results when treated with single-port laparoscopy compared to conventional laparoscopy; 4. Five of the studies involved robotic single-port laparoscopy, which has a better stereoscopic field of view, reduced instrument interference, improved maneuverability and accuracy, and disadvantages such as longer docking time and high cost. These characteristics may be the source of heterogeneity in the study; 5. Although we conducted a comprehensive search of literature related to the study outcome, due to the limited follow-up time of the original study and the absence of relevant outcome indicators, we were unable to conduct a meta-analysis of other surgical outcomes (port hernia), the reproductive endocrine function of the ovary and pregnancy status in single-port versus conventional laparoscopy myomectomy (39); 6. The obstetricians and gynecologists in our included study had rich surgical experience in the operation of single-port laparoscopy, and it should not be ignored that we analyzed the painful differences between the two groups without fully considering the use of analgesic drugs for patients, which may be a potential confounding factor (46).

## 5 Conclusions

In summary, based on the available evidence, surgical outcomes (such as hemoglobin loss, cosmetic results, and length of hospital stay) in the single-port groups are superior to those in the conventional laparoscopy groups in the treatment of uterine fibroids and do not imply any other disadvantages. The operation time, blood loss, postoperative pain, and other aspects of single-port laparoscopy were comparable to those of the traditional groups. However, there was no significant improvement compared to postoperative (fever, first walk time, postoperative exhaust time). Given the limitations of the included studies, it is necessary to confirm and update the results of this analysis with larger, multi-center, well-designed randomized controlled trials in the future.

## Author contributions

YL: Conceptualization, statistics of data, writing drafts. RL: Data survey, data management. XL: Data sorting, polishing the original draft, and collecting data. All authors contributed to this article and approved the version submitted.
