# Supplementary material for: Comparison of efficacy of single-port versus conventional laparoscopic treatment for uterine leiomyoma: a latest meta-analysis
Source: Front Oncol. 2023 Aug 2;13:1192582. doi: 10.3389/fonc.2023.1192582 (PMC10433900; doi:10.3389/fonc.2023.1192582)
Supplement: Supplementary file 1 [file Table_1.doc]

| Author | Year | Single port system | Myoma evacuation | No. of myomas | | Maximal myoma diameter (cm) | | Location of myoma | | Type of myoma | |
| --- | --- | --- | --- | --- | --- | --- | --- | --- | --- | --- | --- |
|  |  |  |  | LESS | MPL | LESS | MPL | LESS | MPL | LESS | MPL |
| Ji Hye Kim[20] | 2022 | Glove port | Morcellation | ＜10:38(82.6);  ≥10: 8(17.4) | ＜10: 39(84.8);≥10: 7(15.2) | 8.3(7.1-9.5) | 8.4(7.6-9.5) | Anterior/ fundus:32 (69.6); Posterior: 9(19.6); Lateral:4 (8.7);Low segment:1 (2.2) | Anterior/ fundus: 29(63); Posterior: 14(30.4); Lateral:3 (6.5);Low segment:0 (0.0) | NA | NA |
| Su Hyeon Choi  [16] | 2019 | Glove port | Retrieval bag | 2.6±2.3 | 4.6±4.1 | 6.3±1.7 | 7.7±2.5 | Anterior:44 (41.9); Anterior fundus:2(1.9); Fundus:14 (13.3); Posterior fundus:2(1.9); Posterior:43 (41.0); | Anterior:105 (42.0); Anterior fundus:4(1.6); Fundus: 37(14.8); Posterior fundus:7(2.8); Posterior:97 (38.8); | Submucosal:4 (3.8);Deep intramural:53 (50.5); Intramural:24 (22.9); Subserosal: 17(16.2); Pedunculated subserosal:2 (1.9); Intraligamentary: 5 (4.8) | Submucosal:5 (2.0);Deep intramural:114 (45.6); Intramural:70 (28.0) Subserosal:52 (20.8); Pedunculated subserosal : 2(0.8); Intraligamentary: 7 (2.8) |
| So Hyun Ahn[15] | 2021 | Multi-channel silicone port | A polyvinyl bag | 2.5± 2.2 | 4.1± 3.7 | 7.7 ± 2.8 | 7.7±3 | NA | NA | FIGO type 2–3:52(57.8); FIGO type 4–5:15(16.7); FIGO type 6–7: 23 (25.6) | FIGO type 2–3:41(45.6); FIGO type 4–5: 24(26.7); FIGO type6–7:25 (27.8) |
| Taejong Song[29] | 2015 | Commercial kit | Scalpel with bag | 1 (1-4) | 1 (1-4) | 6.7±1.8 | 7.2±1.8 | Anterior:17 (34); Posterior: 18 (36); Lateral or fundal:15 (30); Adhesiolysis: 4 (8) | Anterior:16 (32); Posterior: 23(46); Lateral or fundal:11 (22); Adhesiolysis: 2 (4) | NA | NA |
| Tae-Joong Kim  [24] | 2015 | Commercial kit | Scalpel with bag | 1 (1-4) | 1 (1-4) | 6.7±1.8 | 7.2±1.8 | Anterior:17 (34); Posterior: 18 (36); Lateral or fundal:15 (30); Adhesiolysis: 4 (8) | Anterior:16 (32); Posterior: 23 (46); Lateral or fundal:11 (22); Adhesiolysis: 2 (4) | NA | NA |
| Chien-Min Han[18] | 2013 | Glove port | Scalpel | <3 | <3 | 7 (5-7.8) | 6.8 (5.9-8.1) | Anterior:7(70); Posterior:1(10); Fundal: 2 (20) | | NA | NA |
| Gaby N. Moawad  [28] | 2019 | Multi-channel single-port silicone | A specimen bag | 2.4 (2.05) | 4.7 (4.11) | 5.9 (2.02) | 8.3 (3.85) | NA | NA |  |  |
| Seul KiKim  [22] | 2014 | Glove port | Electric morcellator | 1.6±0.8（1-4） | 1.4±0.7（1-5） | 7.3±2.2 （2.9-14.0） | 6.5±2.0 （2.5-10.0） | NA | NA | Intramural (%): 48 (81.4); Subserosal (%):7(11.9); Submucosal: 0; Intraligamentary (%): 4 (6.8) | Intramural (%): 48(81.4); Subserosal (%): 8(13.6); Submucosal: 2 (3.4); Intraligamentary (%) :1 (1.7) |
| Ji YeKim  [21] | 2014 | Commercial kit | Scalpel | NA | NA | 5.0 (2.5-8.0) | 4.8 (2.0-8.0) | Anterior:30 (41.1); Posterior:25 (34.2); Lateral: 6 (8.2); Fundal: 12 (16.4); | Anterior:45 (30.8); Posterior:61 (41.8); Lateral: 23(15.8); Fundal:17 (11.6); | Subserosal: 29 (40.0); Intramural:44 (60.0) | Subserosal:58 (39.7); Intramural:88 (60.3) |
| Dayong  Lee[25] | 2018 | Glove port | Electric morcellator | NA | NA | 6.7 ±1.9 | 6.6 ± 2.2 | NA | NA | FIGO type 1: 16 (53.3); 2: 8 (26.7);3:4 (13.3);4: 2 (6.7) | FIGO type 1: 13 (44.8);2: 13 (44.8);3:3 (10.3); 4: 0 |
| Su Mi Kim[23] | 2015 | Commercial kit | SPLM or CLM | 2.1± 1.5 | 2.2± 2.0 | 7.5± 3.2 | 7.8± 2.6 | Anterior:24 (39.3); Posterior:17 (27.9); Fundal: 15(24.6); Broad ligament:4 (6.6); | Anterior:14 (37.8); Posterior:13 (35.1); Fundal:9 (24.3);Broad ligament:0 (0.0) | Intramural:41 (67.2); Subserosal:15 (24.6); Submucosal:0 (0.0); Intraligamentary: 5 (8.2); | Intramural:24 (64.9); Subserosal:12 (32.4); Submucosal:0 (0.0) ; Intraligamentary: 1 (2.7); |
| Jong Min Baek[17] | 2015 | Commercial kit | SPLM or CLM | 2.1± 1.5 | 2.1± 1.6 | 7.5± 3.2 | 6.5± 2.8 | Anterior:24 (39.3); Posterior:17 (27.9); Fundal: 15(24.6); Broad ligament:4 (6.6); | Anterior:14 (37.8); Posterior:13 (35.1); Fundal: 9(24.3); Broad ligament:0 (0.0) | Intramural:41 (67.2); Subserosal:15 (24.6); Submucosal:0 (0.0); Intraligamentary: 5 (8.2); | Intramural:24 (64.9); Subserosal:12 (32.4); Submucosal:0 (0.0) ; Intraligamentary: 1 (2.7); |
| Shi-Fang Zhou[32] | 2021 | Single-port multichannel cannula | NA | 1.7±1.1 | 1.8± 1.3 | 6.8±1.7 | 7.0±1.8 | NA | NA | Intermural: 161；  Subserosa: 72 | Intermural: 159;  Subserosa: 74 |
| Sa-Ra Lee[26] | 2021 | Multichannel single port | Specimen retrieval bag | 2 (1-34) | 1 (1-7) | 7.6± 2.39 | 8.65± 2.76 | NA | NA | Intramural, n(%):35 (59.32); Submucosal, n (%):4(6.78); Subserosal, n (%): 15 (25.42); Others (intraligamentary, cervical), n (%):5 (8.47) | Intramural, n (%):293 (72.77); Submucosal, n (%):12(2.98); Subserosal, n (%):80(19.85); Others (intraligamentary, cervical), n (%): 18 (4.47) |
| Ju-Hee Kim  [20] | 2021 | Multichannel single port | Specimen retrieval bag | NA | NA | NA | NA | NA | NA | NA | NA |
| Lili Jiang  [19] | 2021 | Glove port | Specimen retrieval bag | 1.70± 1.02 | 1.86 ±1.55 | 7.8 ± 3.10 | 7.40± 2.03 | NA | NA |  |  |
| Wei Zhu  [33] | 2022 | Single-port multichannel cannula | the extraction bag | 2.74 ±1.27 | 2.83± 1.39 | 5.64± 1.57 | 5.88± 1.69 | NA | NA | Intramural myoma24 (68.57)；Subserous myoma11 (31.43) | Intramural myoma 19(63.33)；Subserous myoma11 (36.67) |
| Ying Zhang  [31] | 2021 | Glove port | A specimen bag | 2 ±1.6 | 3 ±1.4 | 6 ±3.4 | 6 ±2.8 | NA | NA | NA | NA |
| Suk Woo Lee[27] | 2017 | Commercial kit | Scalpel with bag | 1.7 ± 0.9(1-4) | 1.6 ± 1.0(1-5) | 7.4(5-13) | 6.8(5-12) | Fundus:17 (17.0); Anterior body: 35(35.0); Posterior body:25 (25.0);Lteral body: 16(16.0); Endometrium: 7 (7.0) | Fundus:16 (23.2); Anterior body: 29(42.0); Posterior body:10 (14.5); Lateral body:10 (14.5); Endometrium: 4 (5.8) | Subserosal:39 (39.0); Intramural:49 (49.0); Submucosal: 7 (7.0); Intraligamentary: 5 (5.0); | Subserosal:5 (50.7); Intramural:28 (40.6); Submucosal:4 (5.8); Intraligamentary: 2 (2.9); |
| Jin-SungYuk[30] | 2015 | Single-port multichannel cannula | Scalpel with bag | 2.5± 1.8 | 2.5± 1.8 | 6.1±2.3 | 6.2±2.0 | Anterior:51 (44.7); Posterior:38 (33.3); Fundal: 20(17.5); Lateral:5 (4.4); | Anterior:53 (46.9); Posterior:34 (30.1); Fundal: 14(12.4); Lateral:12 (10.6); | Subserosal:45 (39.5); Intramural:69 (60.5) | Subserosal:40 (35.4); Intramural:73 (64.6) |

Supply table 1 A table of basic summary clinical information included in the study [No. patients: Number of patients; LESS: laparo-endoscopic single site surgery; MPL: multiple-port laparoscopy；SPLM: Scalpel with bag; CLM : Electric morcellator Data are presented as mean ± standard deviation or n (%) values]
